# Baicalin Ameliorates Cognitive Impairment and Protects Microglia from LPS-Induced Neuroinflammation via the SIRT1/HMGB1 Pathway

**DOI:** 10.1155/2020/4751349

**Published:** 2020-09-22

**Authors:** Yue Li, Taotao Liu, Yitong Li, Dengyang Han, Jingshu Hong, Ning Yang, Jindan He, Ronghui Peng, Xinning Mi, Chongshen Kuang, Yang Zhou, Yongzheng Han, Chengmei Shi, Zhengqian Li, Xiangyang Guo

**Affiliations:** ^1^Department of Anesthesiology, Peking University Third Hospital, No. 49 North Garden Road, Haidian District, Beijing 100191, China; ^2^Department of Anesthesiology, National Cancer Center/National Clinical Research Center for Cancer/Cancer Hospital, Chinese Academy of Medical Sciences and Peking Union Medical College, No. 17 Pan-Jia-Yuan nanli Road, Chaoyang District, Beijing 100021, China

## Abstract

Systemic inflammation often induces neuroinflammation and disrupts neural functions, ultimately causing cognitive impairment. Furthermore, neuronal inflammation is the key cause of many neurological conditions. It is particularly important to develop effective neuroprotectants to prevent and control inflammatory brain diseases. Baicalin (BAI) has a wide variety of potent neuroprotective and cognitive enhancement properties in various models of neuronal injury through antioxidation, anti-inflammation, anti-apoptosis, and stimulating neurogenesis. Nevertheless, it remains unclear whether BAI can resolve neuroinflammation and cognitive decline triggered by systemic or distant inflammatory processes. In the present study, intraperitoneal lipopolysaccharide (LPS) administration was used to establish neuroinflammation to evaluate the potential neuroprotective and anti-inflammatory effects of BAI. Here, we report that BAI activated silent information regulator 1 (SIRT1) to deacetylate high-mobility group box 1 (HMGB1) protein in response to acute LPS-induced neuroinflammation and cognitive deficits. Furthermore, we demonstrated the anti-inflammatory and cognitive enhancement effects and the underlying molecular mechanisms of BAI in modulating microglial activation and systemic cytokine production, including tumor necrosis factor- (TNF-) *α* and interleukin- (IL-) 1*β*, after LPS exposure in mice and in the microglial cell line, BV2. In the hippocampus, BAI not only reduced reactive microglia and inflammatory cytokine production but also modulated SIRT1/HMGB1 signaling in microglia. Interestingly, pretreatment with SIRT1 inhibitor EX-527 abolished the beneficial effects of BAI against LPS exposure. Specifically, BAI treatment inhibited HMGB1 release via the SIRT1/HMGB1 pathway and reduced the nuclear translocation of HMGB1 in LPS-induced BV2 cells. These effects were reversed in BV2 cells by silencing endogenous *SIRT1*. Taken together, these findings indicated that BAI reduced microglia-associated neuroinflammation and improved acute neurocognitive deficits in LPS-induced mice via SIRT1-dependent downregulation of HMGB1, suggesting a possible novel protection against acute neurobehavioral deficits, such as delayed neurocognitive recovery after anesthesia and surgery challenges.

## 1. Introduction

Delayed neurocognitive recovery (dNCR), formally known as postoperative cognitive decline (POCD) [1], is characterized by a decline in cognitive function that occurs in patients up to 30 days after anesthesia and surgery. The development of dNCR has been reported to be associated with daily function impairment and increased morbidity and mortality leading to an economic burden on healthcare resources [2]. Previous studies including ours have found that neuroinflammation has a key role in the pathophysiology of dNCR [3, 4]. Blood-borne factors, as well as the proinflammatory systemic milieu, can adversely affect the central nervous system (CNS) function by directly affecting synaptic plasticity and cognitive function [5]. Systemic alarmins and cytokines such as interleukin- (IL-) 1*β*, tumor necrosis factor- (TNF-) *α*, and high-mobility group box 1 (HMGB1) protein trigger neuroinflammation after peripheral surgery in mouse models [6]. Activated microglia, which secrete proinflammatory factors, have been well documented to play an important role in CNS dyshomeostasis. Lipopolysaccharide (LPS) can trigger a systemic inflammatory response via activation of pattern recognition receptors (including toll-like receptors), cytokines, and oxidative stress pathways. We chose the intraperitoneal injection of LPS, which is a more clinical method, rather than intracranial LPS injection. This method is an effective animal model for dNCR [7, 8].

Silent information regulator 1 (SIRT1) is an NAD-dependent nuclear histone deacetylase that plays important roles in maintaining cellular homeostasis including metabolism, cell survival, and insulin resistance [9]. SIRT1 also plays a significant role in regulating normal brain functions such as plasticity and memory [10, 11]. Furthermore, it has been demonstrated that SIRT1 deacetylates inflammation-related transcription factors such as NF-*κ*B to reverse inflammation [12]. Decreased expression levels of SIRT1 have been observed in the hippocampus of aged dNCR model mice [13]. dNCR model mice respond to therapies by upregulating SIRT1 expression in the hippocampus, as shown by a neuroprotective treatment of aged mice with SIRT1 activators [14, 15]. Recent reports have shown that SIRT1 is involved in protein quality control, as SIRT1 suppressed aggregation of tau proteins [16] and HMGB1 transcription [17] by direct deacetylation regulation.

HMGB1, a damage-associated molecular pattern (DAMP) family member, is a ubiquitous nuclear protein that contributes to the chromatin architecture in physiological conditions [18]. Under pathophysiological conditions, HMGB1 acts as a pathogenic inflammatory factor. Its translocation from the nucleus to the extracellular space is mediated by acetylation; it is released by glia and neurons upon inflammasome activation via an N-methyl D-aspartate receptor subtype 2B- (NR2B-) mediated mechanism [19]. Extracellular HMGB1 mediates sterile inflammation and induces cells to release inflammatory cytokines by binding to receptor for advanced glycation end products (RAGE) and toll-like receptor 4 (TLR4). This binding then activates the NF-*κ*B pathway directly or indirectly via the phosphoinositide 3-kinase (PI3K) or mitogen-activated protein kinases (MAPKs) pathway to promote inflammation and facilitate the upregulation of HMGB1 [20]. Moreover, previous reports have demonstrated that HMGB1 serves as an adverse factor in memory impairment, neurodegeneration, and neuroinflammatory progression.

Baicalin (BAI) is an important flavonoid compound, which is responsible for most of the biological effects of *Scutellaria baicalensis* [21]. BAI is a Food and Drug Administration- (FDA-) approved pharmaceutical food flavonoid. BAI could penetrate the blood-brain barrier (BBB) and distribute into the cerebrospinal fluid, which tends to accumulate in the striatum, thalamus, and hippocampus [22]. Additionally, BAI administration increased the expression of GABA_A_ receptor and *α*-amino-3-hydroxy-5-methyl-4-isoxazolepropionic acid (AMPA) receptor and decreased the activation of NLRP3 inflammasome in the hippocampus [21]. A number of studies have shown that BAI ameliorates various inflammatory diseases and prevents neurodegenerative diseases through various mechanisms [23–26], including suppression of oxidative stress, apoptosis, and inflammation, neurogenesis stimulation, and promotion of brain-derived neurotrophic factor (BDNF) expression. Moreover, the neuroprotective effects of BAI in neurological disorders like depressive-like behaviors [12] or oxidative stress injury of SH-SY5Y cells [27] have been linked to SIRT1 activation. BAI dramatically suppressed the HMGB1 protein expression in the hippocampal tissues of chronic unpredictable mild stress- (CUMS-) induced mice [28]. BAI also mitigates long-term LPS-evoked depression model symptoms by alleviating microglial activation [29, 30]. To date, no studies have elucidated whether BAI affects the acute LPS-induced microglial activation and inflammatory response in a SIRT1-dependent manner. In the current study, we explored the cognitive-improving effects of BAI upon single administration of LPS induction. We also investigated the underlying neuroprotective mechanisms with emphasis on improving microglial activation and neuroinflammation through SIRT1 upregulation and repression of the HMGB1 signaling pathway, thereby suggesting a new role of BAI in mediating cognition decline.

## 2. Materials and Methods

### 2.1. Drugs and Reagents

**Figure 1:**
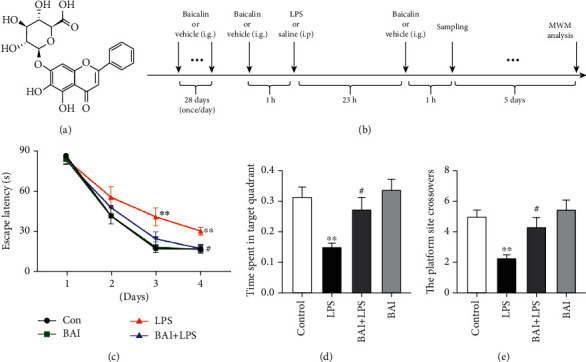
BAI ameliorated learning and memory deficits in LPS-induced mice. Mice were divided into four groups: control, LPS, BAI+LPS, and BAI. (a) Chemical molecular formula of BAI. (b) Schematic timeline of the experimental procedure. (c) The escape latency of LPS-induced mice in the MWM navigation test. (d) Analysis of the time spent in each quadrant during the probe trial of the MWM. (e) The platform crossing times during the probe trial of the MWM test. Data are shown as means ± SEM (*n* = 10–12 per group) and were compared by repeated measure 2-way ANOVA with Bonferroni post hoc analysis. ^∗^*P* < 0.05, ^∗∗^*P* < 0.01, control vs. LPS; ^#^*P* < 0.05, BAI+LPS vs. LPS. BAI, baicalin; MWM, Morris water maze; LPS, lipopolysaccharide; SEM, standard error of the mean.

BAI (Figure 1(a); 98% purity, CAS number 21967-41-9) was bought from Tauto Biotech (Shanghai, China). LPS from *Escherichia coli* O111:B4 and protease and phosphatase inhibitor tablets were purchased from Sigma-Aldrich (St. Louis, MO, USA). EX-527, also known as Selisistat, was purchased from Selleck (Houston, TX, USA). Rabbit anti-SIRT1, rabbit anti-HMGB1, rabbit antiacetylated lysine, and mouse anti-GFAP were from Cell Signaling Technology (Beverly, MA, USA); mouse anti-TNF-*α* and mouse anti-HMGB1 were from Santa Cruz Biotechnology (Santa Cruz, CA); rabbit anti-IL-1*β*, goat antimouse IgG H&L (Alexa Fluor® 594), and goat antirabbit IgG H&L (Alexa Fluor® 594/488) were from Abcam (Cambridge, MA, USA); rabbit anti-Iba-1 was from Wako (Rosemont, IL, USA); mouse anti-LaminB1, mouse anti-GAPDH, and mouse anti-*β*-actin were from Proteintech Group (Wuhan, China). Protein A/G-coupled magnetic beads were from Invitrogen (Carlsbad, CA, USA).

### 2.2. Animals

Adult male C57BL/6 mice (10–11weeks, 21–23 g) were purchased from the Department of Laboratory Animal Research Center, Peking University, and maintained under standard laboratory conditions. All mice were acclimated for 1 week in a controlled environment of 24 ± 1°C, 60% humidity, and 12 : 12 h light-dark cycle with free access to food and water. All animal experiments were conducted in accordance with the guidelines for experimental animal use, and the protocol was approved by the animal ethics committee of Peking University Health Science Center.

### 2.3. Experimental Model and Drug Administration

After the acclimation, mice were randomly assigned into six groups (*n* = 8–12 in each group): (i) control (0.9% NaCl, intraperitoneal injection i.p.), (ii) LPS (2 mg/kg i.p.), (iii) LPS+BAI (2 mg/kg LPS i.p.+103 mg/kg BAI, intragastric gavage i.g., daily), (iv) BAI alone (103 mg/kg), (v) BAI+LPS+EX-527 (a selective SIRT1 inhibitor, 5 mg/kg/day, every 2 days for a total of 7 days before LPS and 5 mg/kg/day after LPS), and (vi) LPS+EX-527. We chose this LPS dose based on previous studies in which LPS at 2 mg/kg reliably induced sickness behavior and learning deficits in C57BL/6 J mice [31, 32]. The chemical structure of BAI is shown in Figure 1(a). BAI, suspended in water containing 0.3% carboxymethylcellulose sodium (CMC-Na), was administered daily by intragastric gavage, which was started 4 weeks before LPS treatment and continued for another 6 days for a total of 34 days (Figure 1(b)). The behavioral tests were performed 24 h after the LPS administration. The doses of BAI and EX-527 were chosen based on earlier reports in which BAI significantly improved learning and memory deficits of APP/PS1 mice and inhibited the expression of SIRT1, respectively [33, 34].

### 2.4. Morris Water Maze (MWM)

The MWM, which is a hippocampus-dependent spatial navigation and reference memory test for rodents, was conducted as described previously with minor modifications [35]. The MWM (Sunny Instruments Co. Ltd., Beijing, China) was performed in a white circular tank (120 cm in diameter and 50 cm in height) filled with white water maintained at 23 ± 1°C. The maze was divided into four quadrants. An invisible fixed platform (10 cm in diameter) was immersed 1 cm below the water surface in the target quadrant. Mice were gently placed in one quadrant facing the wall of the MWM pool. The mice were allowed to swim for 90 s to locate the hidden platform during each trial. When successful, the mouse was allowed to stay for 10 s on the platform. If unsuccessful within the time limit, the mouse was guided to the platform for 10 s. A 3–5 min interval was allowed between each trial. The time spent to locate the platform, swimming speed, and distance were recorded by a video camera. Four spatial acquisition trials were performed with each mouse per day. Latency (s) was measured to assess the time taken to reach the hidden platform for four consecutive days. On the following day, we performed the probe test for the evaluation of memory consolidation by removing the platform and allowing the animal to swim freely for 90 s. The number of crossings over the previously hidden platform and the time spent in the specific target quadrant were recorded.

### 2.5. Immunohistochemistry and Immunofluorescence

Mice were transcardially perfused with phosphate-buffered saline (PBS, pH 7.3), followed by 50 ml of 4% paraformaldehyde in PBS. Tissues were processed for paraffin embedding and 6-*μ*M sections were prepared. The paraffin sections were deparaffinized, rehydrated, and blocked. For immunohistochemistry, the sections were incubated with anti-Iba-1 (1 : 500) and anti-GFAP (1 : 200) overnight at 4°C. Subsequently, the sections were washed with PBS and incubated with goat antirabbit or antimouse secondary antibodies at room temperature for 30 min. Next, the 3, 3-diaminobenzidine color reaction was performed. For immunofluorescence, the sections were incubated with antibodies against HMGB1, Iba-1, and SIRT1. Subsequently, the sections were incubated with Alexa Fluor® 594 or Alexa Fluor® 488 secondary antibodies for 1 h. The slides were imaged with Leica microsystems (Wetzlar, Germany). Immunohistochemical sections were evaluated by assessing the signal intensity (five grades) [20]: 0, no detectable stained cells; 1, very low staining of cells; 2, moderate density of positive cells; 3, submaximal density of positive cells; and 4, the highest density of positive cells.

### 2.6. Cell Culture

Murine BV2 microglial cells were purchased from China Infrastructure of Cell Line Resource (Beijing, China) and cultured in Dulbecco's modified Eagle medium (DMEM) (Gibco, Invitrogen) supplemented with 10% fetal bovine serum (FBS) (Gibco, Invitrogen) at 37°C in a humidified incubator chamber under an atmosphere of 5% CO_2_. The cells were pretreated with BAI (40 *μ*M) for 2 h followed by incubation with LPS (100 ng/ml) for 24 h.

For small interfering RNA (siRNA) transfection, microglia were transfected with *SIRT1*-specific siRNA (GenePharma, Shanghai, China) or negative control siRNA for 6 h using RNAiMAX (Invitrogen) with Opti-MEM Reduced-Serum Medium (Gibco, Invitrogen). Then, cells were maintained in DMEM supplemented with 10% FBS at 37°C in a humidified incubator chamber under an atmosphere of 5% CO_2_ for an additional 36 h. Next, the microglia were exposed to LPS (100 ng/ml) with or without BAI (40 *μ*M). Western blot analysis was used to evaluate the siRNA efficiency.

### 2.7. Cell Viability

The Cell Counting Kit-8 (CCK8, Dojindo, Kumamoto, Japan) was used to assess the viability of BV2 cells. Cells were incubated in DMEM+10% FBS in 96-well plates at a density of 1 × 10^4^ cells/well in a 5% CO_2_ incubator and treated with various concentrations of BAI for 24 h. Thereafter, 10 *μ*l of CCK8 reagent was added, and the plates were incubated at 37°C for 2 h in a 5% CO_2_ incubator. Absorbance was measured at 450 nm.

### 2.8. Determination of Extracellular HMGB1 Levels

The HMGB1 levels released into the culture media were determined based on previously published methods [36]. Equal aliquots of conditioned media from equal numbers of BV2 cells that were treated with the indicated reagents for the indicated durations were used to measure the amount of HMGB1 secreted into the culture media. Eighty percent ice-cold acetone was used to precipitate the proteins in the conditioned media at −20°C for 1 h. After centrifugation at 16000 × *g* for 10 min at 4°C, the protein pellets were washed with 80% ice-cold acetone. The pellets were then resuspended in SDS-PAGE sample buffer, and the levels of HMGB1 were determined by immunoblot analysis.

### 2.9. Western Blot Analysis

Hippocampal tissues of LPS-treated mice and treated BV2 cells were lysed with RIPA buffer containing protease and phosphatase inhibitors. The extracted proteins were then centrifuged at 15000 × *g* for 10 min at 4°C, and the concentration of the supernatants was quantified by the bicinchoninic acid (BCA) protein kit according to the manufacturer's instructions (Beyotime, Shanghai, China). Equal amounts of protein were separated by 10% SDS-PAGE and transferred onto polyvinylidene difluoride (PVDF) membranes. After blocking with 5% FBS in TBS-T (TBS containing 0.1% Tween 20), the membranes were incubated at 4°C overnight with the following primary antibodies: anti-SIRT1 (1 : 1000), anti-HMGB1 (1 : 1000), anti-TNF-*α* (1 : 1000), anti-IL-1*β* (1 : 1000), anti-Iba-1 (1 : 1000), anti-GFAP (1 : 1000), anti-LaminB1 (1 : 2000), anti-GAPDH (1 : 5000), and anti-*β*-actin (1 : 5000). After incubation with secondary antibodies for 1 h at room temperature, the membranes were exposed to enhance chemiluminescence and then analyzed using the Gel-Pro analyzer software (Rockville, MD, USA).

### 2.10. Coimmunoprecipitation

Whole-cell lysates were precleared with protein A/G-coupled magnetic beads and then incubated with 1 mg of anti-HMGB1 antibodies overnight at 4°C. Subsequently, the mixture was incubated for 4 h with protein A/G magnetic beads. Samples were then extensively washed four times with PBS and then boiled. The immunoblot analysis was performed with antiacetylated lysine antibodies.

### 2.11. Immunocytochemistry

BV2 cells were washed with cold PBS and fixed with 4% paraformaldehyde solution for 10 min and then washed thrice with 1×PBS solution for 15 min. Subsequently, the cells were permeabilized with 0.5% Triton X-100 for 5 min and then blocked with PBS containing 10% BSA. The slides were incubated with anti-HMGB1 (1 : 100) overnight at 4°C. After three washes, the cells were incubated with Alexa Fluor® 594 secondary antibodies at 37°C for 1 h and the nuclei were stained with DAPI for 10 min. Fluorescent images were obtained using a TCS SP8 X confocal fluorescence microscope (Mannheim, Germany).

### 2.12. Cytoplasmic and Nuclear Protein Extraction

Extraction was strictly performed according to the manufacturer's instructions for nuclear and cytoplasmic protein extraction kits (Beyotime Institute of Biotechnology, Shanghai, China). BV2 cells were lysed in cytosol fractionation buffer for 5 min and then centrifuged at 10000 × *g* at 4°C for 1 min, to obtain the supernatant containing the cytosolic fraction. The pellet was lysed in nuclear fractionation buffer on ice for 15 min. The nuclear fraction (supernatant) was obtained by centrifugation at 10000 × *g* at 4°C for 15 min.

### 2.13. Statistical Analysis

Data from at least three experiments were used for analyses with the GraphPad Prism 8.0 Software. Quantitative data are shown as means ± SEM. One-way ANOVA followed by Bonferroni post hoc test was used for multiple comparisons. The MWM data were analyzed using two-way ANOVA with repeated measures followed by a Bonferroni post hoc test to analyze the difference in escape latency between each group. *P* < 0.05 was considered significant.

## 3. Results

### 3.1. BAI Ameliorates LPS-Induced Spatial Learning and Memory Impairment

Previous studies have demonstrated that intraperitoneal administration of LPS leads to spatial learning and memory deficits [7, 31]. Therefore, the protective effects of BAI on LPS-induced cognitive deficits were examined in this well-established mouse model. There were no significant differences in swimming speed among the four groups (data not shown). LPS-treated mice showed significantly enhanced escape latencies compared with the control mice on days 3 and 4 (Figure 1(c)), suggesting that LPS caused spatial learning impairments. Pretreating LPS-stimulated mice with BAI significantly attenuated the escape latencies on day 4 of training (Figure 1(c)). There were no significant differences in latencies between the control and BAI groups. In the probe test, the frequency of crossing the platform and the time spent in the target quadrant were both significantly increased in the LPS-injected mice pretreated with BAI relative to the LPS group (Figures 1(d) and 1(e)), suggesting that memory impairments after LPS exposure can be remarkably alleviated by BAI administration. Collectively, these findings indicated that BAI treatment ameliorated LPS-induced spatial learning and memory impairments.

### 3.2. BAI Inhibits LPS-Stimulated Glial Activation and Cytokine Release in the Hippocampus

**Figure 2:**
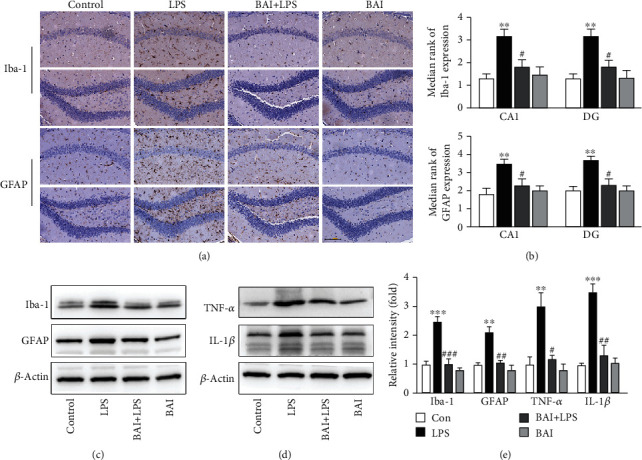
BAI attenuated LPS-induced activated glial cells and the inflammatory response in the mouse hippocampus. (a) Representative photomicrographs of the immunohistochemical analysis of microglia (Iba-1-positive cells) and astrocytes (GFAP-positive cells) in the hippocampal CA1 and DG subregions of mice 24 h after LPS administration and (b) their statistical analysis (scale bars: 100 *μ*M; *n* = 6/group). (c) Immunoblotting was used to determine the Iba-1 and GFAP protein levels in hippocampal tissue lysates. (d) IL-1*β* and TNF-*α* expression in the hippocampus was determined by immunoblotting 24 h after LPS and BAI administration. (e) Quantification of the data shown in c and d. Data are presented as the mean ± SEM (*n* = 6 per group). ^∗∗^*P* < 0.01, ^∗∗∗^*P* < 0.001, control vs. LPS; ^#^*P* < 0.05, ^##^*P* < 0.01, ^###^*P* < 0.001, BAI+LPS vs. LPS.

Evidence has shown that microglia and astrocytes are associated with proinflammatory responses upon surgical trauma. Inhibition of microglia can lead to a reduction in proinflammatory cytokines including TNF-*α* and IL-1*β*, which are all important factors in neuroinflammation. Thus, we examined the effects of BAI on LPS-induced microglial and astrocyte activation *in vivo*. The LPS-injected mice showed significant activation of microglia (Iba-1) and astrocytes (GFAP) in the CA1 and DG subregions of the hippocampus (Figures 2(a) and 2(b)). In contrast, cotreatment with BAI remarkably inhibited microglial and astrocyte activation, as shown by immunohistochemistry (Figures 2(a) and 2(b)). Similarly, the western blot results showed that LPS significantly increased the expression of Iba-1 and GFAP in the hippocampus, whereas BAI suppressed this expression (Figures 2(c) and 2(e)). Furthermore, compared with the control mice, LPS-injected mice exhibited a significant increase in IL-1*β* and TNF-*α* levels in the hippocampus (Figures 2(a) and 2(e)). Similar to the previous results, the increased levels of IL-1*β* and TNF-*α* were largely reduced by BAI coadministration (Figures 2(a) and 2(e)). These results further support the notion that BAI modulates LPS-induced glial activation as well as the levels of the proinflammatory cytokines, IL-1*β* and TNF-*α*, in the mouse hippocampus.

### 3.3. BAI Upregulates SIRT1 and Downregulates HMGB1 Expression in Microglia In Vivo and In Vitro

**Figure 3:**
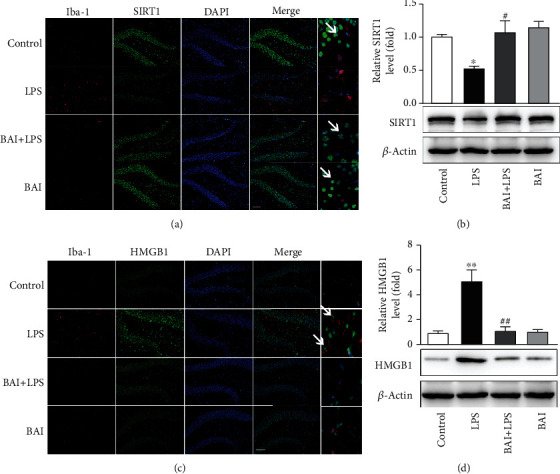
BAI upregulated SIRT1 expression and downregulated HMGB1 expression in mice with hippocampal impairment. (a) Immunofluorescence of SIRT1 (green) in microglial cells (Iba-1+; red) in the mouse hippocampus 24 h after LPS exposure. *Arrows* point to SIRT1-positive microglial cells (Iba-1+). (b) Western blot analysis of SIRT1 expression in mice subjected to LPS and BAI. (c) Immunofluorescence of HMGB1 (green) in microglial cells (Iba-1+; red) in the hippocampus 24 h after LPS exposure. *Arrows* point to HMGB1-positive microglial cells (Iba-1+). (d) Western blot analysis of HMGB1 expression in mice subjected to LPS and BAI. Data are expressed as the mean ± SEM (*n* = 4–5). ^∗^*P* < 0.05, ^∗∗^*P* < 0.01, control vs. LPS; ^#^*P* < 0.05, ^##^*P* < 0.01, BAI+LPS vs. LPS. SIRT1, silent information regulator 1; HMGB1, high-mobility group box protein 1.

A growing body of evidence supports an important role for SIRT1 in cognitive dysfunction and the inflammatory response [14, 37]. To examine whether the SIRT1 protein level is modulated in the LPS-exposed mouse hippocampus, we immunostained SIRT1 in microglia (Iba-1^+^) in hippocampal slices of LPS-treated mice. We observed impaired SIRT1 protein expression in microglia exposed to LPS, which was restored by BAI treatment (Figure 3(a)). These results were further confirmed by western blotting (Figure 3(b)). HMGB1 may play a key role in triggering the inflammatory cascade leading to CNS dysfunction. Elevated HMGB1 levels after surgery are associated with compromised blood-brain barrier (BBB) and subsequent neuroinflammation [38]. Immunofluorescence staining of Iba-1 and HMGB1 as well as immunoblotting for HMGB1 detection demonstrated that HMGB1 largely increased in the activated microglia in the hippocampus 1 day after LPS exposure (Figures 3(c) and 3(d)). In contrast, HMGB1 expression was inhibited by BAI supplementation (Figures 3(c) and 3(d)). These *in vivo* results indicate that BAI upregulated SIRT1 and downregulated HMGB1 expression in hippocampal microglia.

**Figure 4:**
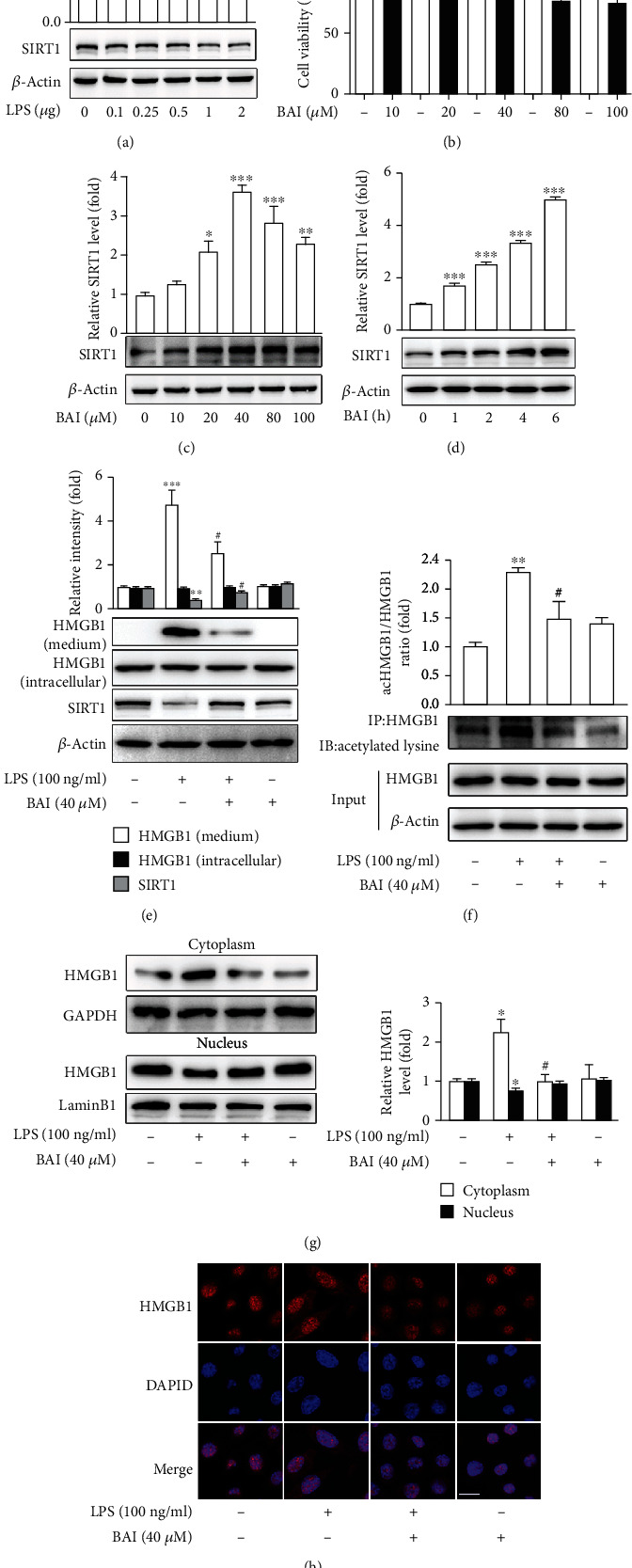
BAI significantly increased SIRT1 expression and decreased HMGB1 release in LPS-induced BV2 microglia. (a) BV2 cells were stimulated with various concentrations of LPS (0.1, 0.25, 0.5, 1, and 2 *μ*g/ml) for 24 h, followed by western blot analysis of SIRT1 protein. (b) BV2 cells were cultured with the indicated BAI concentrations (10, 20, 40, 80, and 100 *μ*M) for 24 h, and cell viability was determined by CCK8. (c, d) BV2 cells were cultured with different BAI concentrations for 24 h (c) or with 40 *μ*M BAI for different durations (1, 2, 4, and 6 h) (d), and SIRT1 levels were analyzed by immunoblotting. (e) BV2 cells were pretreated with 40 *μ*M BAI for 2 h, followed by 100 ng/ml LPS stimulation for 24 h. Western blot analysis of the levels of SIRT1, secreted HMGB1, and intracellular HMGB1 was performed. (f) BV2 cells were preincubated with 40 *μ*M BAI for 2 h and then stimulated with 100 ng/ml LPS for 4 h. Acetylated lysine and HMGB1 expression levels were determined by immunoprecipitation. (g) BV2 cells were pretreated with 40 *μ*M BAI for 2 h, followed by 100 ng/ml LPS treatment for 6 h. The nucleocytoplasmic translocation of HMGB1 was determined by western blot analysis. GAPDH and LaminB1 were used as loading controls for the cytoplasmic and nuclear fractions, respectively. (h) Immunofluorescence of the HMGB1 (red) cytosolic translocation was conducted with confocal scanning microscopy. Scale bar = 10 *μ*M. All data were normalized to the control group and are presented as means ± SEM (*n* = 3). ^∗^*P* < 0.05, ^∗∗^*P* < 0.01, ^∗∗∗^*P* < 0.001, control vs. LPS; ^#^*P* < 0.05, BAI+LPS vs. LPS.

According to the above results, glial cells, particularly microglia, are LPS-responsive cells in the CNS. To further investigate the protective effect of BAI against LPS-induced neuroinflammation, BV2 microglia were used *in vitro*. To investigate whether SIRT1 is involved in LPS toxicity in BV2 microglia, we compared SIRT1 protein levels in control cells and cells treated with different LPS concentrations (0.1, 0.25, 0.5, 1, and 2 *μ*g/ml; Figure 4(a)). SIRT1 protein level decreased after the LPS challenge (Figure 4(a)). To determine the optimal BAI concentration, we examined the viability of BV2 cells treated with vehicle (1% DMSO) or various BAI concentrations for 24 h (Figure 4(b)). Treatment with up to 40 *μ*M BAI did not elicit cytotoxic effects on BV2 cells (Figure 4(b)). Next, we examined the effect of BAI treatment on SIRT1 expression. BAI (20, 40, 80, and 100 *μ*M) obviously upregulated SIRT1 expression in BV2 cells (Figure 4(c)), in a time-dependent manner (Figure 4(d)). Thus, we selected 40 *μ*M BAI as the optimal concentration for subsequent experiments.

BV2 microglia were treated with vehicle (1% DMSO) or 40 *μ*M BAI for 2 h, followed by 100 ng/ml LPS or PBS for 24 h. Consistent with the *in vivo* experiments, SIRT1 expression was markedly low in LPS-exposed BV2 cells (Figure 4(e)), and this effect was reversed by BAI treatment (Figure 4(e)). Additionally, BAI+LPS cotreatment greatly downregulated the level of HMGB1 release compared with the LPS-treated BV2 cells (Figure 4(e)). HMGB1 has been reported to translocate from the nucleus to the cytoplasm in response to inflammatory signals such as LPS. Furthermore, inflammation-induced acetylation of HMGB1 is a vital regulator of its release to the extracellular compartment, where it is a substrate of SIRT1 [18]. Therefore, we examined whether BAI affects this LPS-induced HMGB1 acetylation and translocation in BV2 microglial cells by western blot and immunofluorescence analyses (Figures 4(f)–4(h)). HMGB1 translocation to the cytoplasm increased in cells exposed to LPS; however, these changes were restored by BAI treatment (Figures 4(g) and 4(h)). Furthermore, when BV2 cells were stimulated with LPS for 4 h, the level of acetylated HMGB1 was significantly enhanced; however, BAI cotreatment with LPS markedly reversed this effect (Figure 4(f)). Our data confirmed that BAI treatment induced SIRT1 expression and suppressed HMGB1 acetylation and translocation, thereby counteracting the effects of LPS in mice and BV2 cells.

### 3.4. BAI Neuroprotection against LPS-Induced Cognitive Deficits Is SIRT1 Dependent

**Figure 5:**
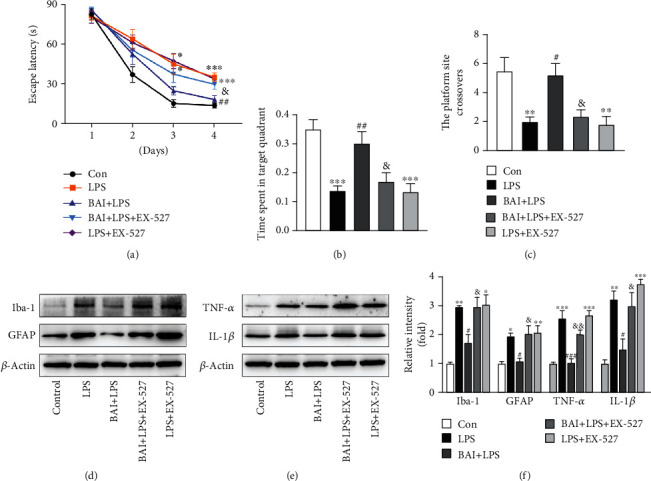
BAI-improved neuroinflammation and neurocognitive impairment induced by exposure to LPS in mice are SIRT1 dependent. Mice were divided into five groups: control, LPS, BAI+LPS, BAI+LPS+EX-527, and LPS+EX-527. (a) Latency to the platform during spatial working memory testing. (b) Time spent in the target quadrant during probe testing. (c) Platform-site crossings during the probe trial. Data are expressed as the mean ± SEM (*n* = 8–10). (d) Western blot analysis of Iba-1 and GFAP in the hippocampus following LPS and BAI treatment, with or without EX-527 treatment. (e) Immunoblots of the inflammatory cytokines, IL-1*β* and TNF-*α*. (f) Quantification of the data shown in d and e. Data are expressed as the mean ± SEM (*n* = 5/group). ^∗^*P* < 0.05, ^∗∗^*P* < 0.01, ^∗∗∗^*P* < 0.001, control vs. LPS or LPS+EX-527; ^#^*P* < 0.05, ^##^*P* < 0.01, ^###^*P* < 0.001, BAI+LPS vs. LPS; ^&^*P* < 0.05, ^&&^*P* < 0.01, BAI+LPS vs. BAI+LPS+EX-527. EX-527, a specific SIRT1 inhibitor.

To explore whether the neuroprotective effect of BAI is SIRT1 dependent, we pretreated mice with EX-527, a specific SIRT1 inhibitor. The LPS group and LPS+EX-527 group exhibited longer escape latencies, fewer platform crossings, and shorter target quadrant times than the control mice on days 3 and 4 (Figures 5(a)–5(c)). There was no difference between the LPS group and the LPS+EX-527 group (Figures 5(a)–5(c)). In contrast, in the BAI+LPS group, the latencies to find the platform on day 4 of training were shorter and the frequency of crossing the platform and the time spent in the target quadrant significantly increased (Figures 5(a)–5(c)). Interestingly, EX-527 treatment affected the BAI inhibitory effect on cognition decline. Administration of EX-527 prior to LPS and BAI treatment abolished the protective effects of BAI (Figures 5(a)–5(c)). Pretreating BAI+LPS mice with Ex-527 extended their latency to find the platform on day 4 of training (Figure 5(a)). In the probe test, the platform-crossing times and the time spent in the target quadrant were significantly shorter in the BAI+LPS+EX-527 group than in the BAI+LPS group (Figures 5(b) and 5(c)). These results indicate that BAI pretreatment was effective in improving spatial learning and memory of LPS-treated mice in a SIRT1-dependent manner.

We then examined the effect of BAI-mediated SIRT1 activation on inflammation *in vivo* using LPS-induced mice. The results showed that the BAI-inhibited glial cell activation was abolished by EX-527 treatment. Compared with the LPS and LPS+EX5-27 groups, BAI administration decreased the expression of Iba-1 and GFAP in the hippocampus, whereas pretreatment of the BAI+LPS-treated mice with EX-527 increased their expression (Figures 5(d) and 5(f)). Furthermore, we observed that the expression levels of TNF-*α* and IL-1*β* in the hippocampus were increased in the LPS and LPS+EX-527 groups relative to the control group, and there was no difference in the expression of these proteins between the LPS and LPS+EX-527 groups (Figures 5(e) and 5(f)). In contrast, BAI significantly suppressed TNF-*α* and IL-1*β* expression relative to mice subjected to LPS, which was reversed by topical EX-527 treatment (Figures 5(e) and 5(f)). Taken together, our results suggest that BAI effectively ameliorated the memory deficits and inhibited microglial activation and proinflammatory cytokines' expression induced by systemic LPS treatment through a SIRT1-dependent mechanism in the hippocampus.

### 3.5. HMGB1, A SIRT1 Downstream Molecule, Participates in the Microglial Protective Action of BAI against LPS-Induced Neuroinflammation

**Figure 6:**
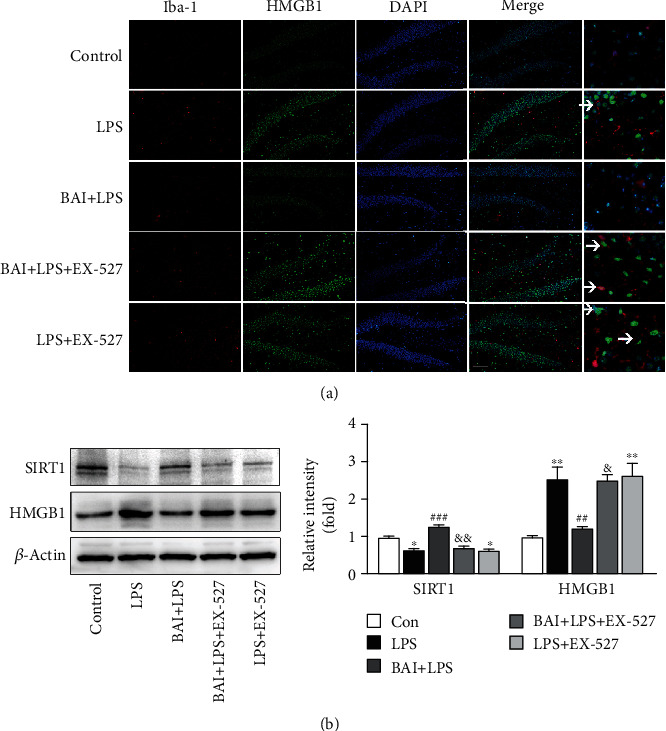
BAI suppressed the HMGB1 pathway by elevating SIRT1 in the hippocampus of LPS-induced mice. (a) HMGB1 immunofluorescence (green) in microglial cells (Iba-1+; red) counterstained with DAPI (blue), following LPS and BAI treatment; scale bars = 100 *μ*M. *Arrows* point to HMGB1-positive microglial cells (Iba-1+). (b) Western blot analysis confirming the effect of BAI and EX-527 on SIRT1 and HMGB1 protein expression in LPS-induced mice. Data are expressed as the mean ± SEM (*n* = 5/group). ^∗^*P* < 0.05, ^∗∗^*P* < 0.01, control vs. LPS or LPS+EX-527; ^##^*P* < 0.01, ^###^*P* < 0.001, BAI+LPS vs. LPS; ^&^*P* < 0.05, ^&^^&^*P* < 0.01, BAI+LPS vs. BAI+LPS+EX-527.

HMGB1 as a downstream mediator of systemic inflammation has opened a new avenue for the development of anti-inflammatory therapeutics. SIRT1 dissociates from HMGB1 during translocation from the nucleus to the cytosol and promotes the HMGB1 acetylation [39, 40]. SIRT1-dependent HMGB1 deacetylation regulates microglial polarization and the subsequent neuroinflammatory response [41]. To analyze whether the BAI-mediated deactivation of the HMGB1 signaling pathway is SIRT1 dependent, we used EX-527, *in vivo* (Figures 6(a) and 6(b)). The immunoblot results indicated that BAI treatment reduced LPS-induced HMGB1 expression, whereas SIRT1 inhibition in the BAI+LPS group resulted in upregulated HMGB1 expression (Figure 6(b)). Similarly, we conducted a double immunofluorescent staining to assess HMGB1 expression in microglia (Iba-1^+^) in the hippocampus after LPS exposure (Figure 6(a)). The results showed that the LPS-induced increase in HMGB1 protein expression in microglia was SIRT1 dependent and that BAI significantly decreased the expression of HMGB1 and Iba-1 proteins in hippocampal microglia from LPS-treated mice (Figure 6(a)). This finding suggests that BAI increased the level of SIRT1 and inhibited the activation of the HMGB1 signaling pathway, which alleviated the activation of microglia and mitigated neuroinflammation.

**Figure 7:**
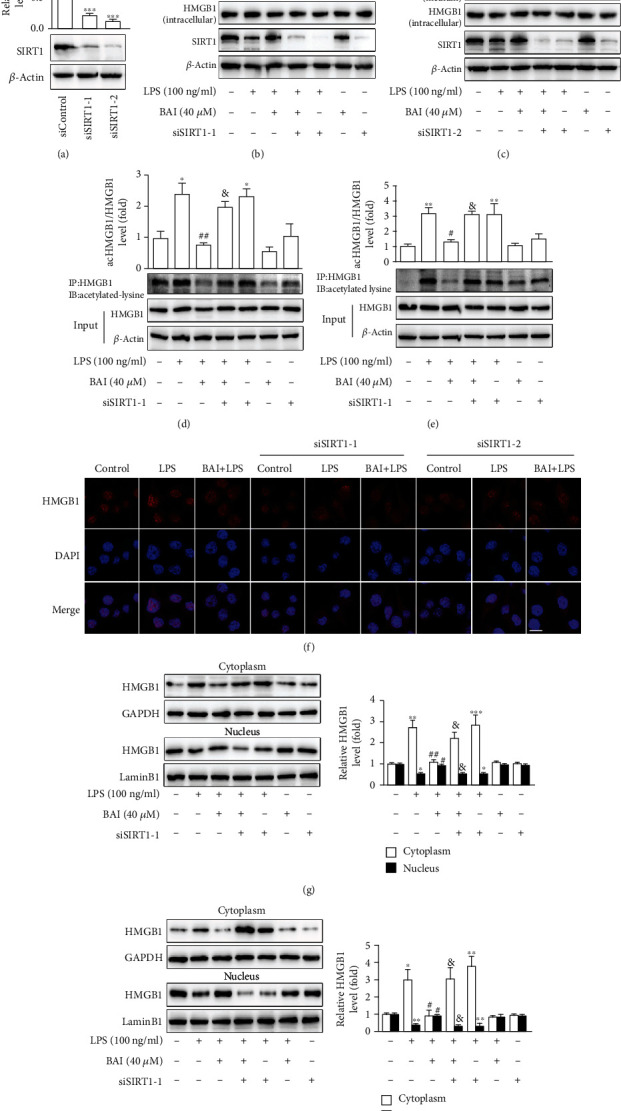
SIRT1 is required for BAI inhibition of LPS-induced HMGB1 release from BV2 microglial cells. BV2 cells were transfected with *SIRT1*-targeted siRNA-1, siRNA-2, or control siRNA. (a) SIRT1 protein levels in siRNA-1 or siRNA-2-transfected BV2 cells were measured by immunoblotting. (b, c) After transfection for 36 h, cells were exposed to LPS in the presence or absence of BAI for 24 h, and the expression of SIRT1, secreted HMGB1, and intracellular HMGB1 was measured by western blotting. (d, e) After transfection, BV2 cells were cultured in LPS medium alone or with 40 *μ*M BAI for 4 h. Coimmunoprecipitation analysis showed the level of acetylated HMGB1. (f) Cells transfected with SIRT1-targeted siRNA-1 or siRNA-2 for 36 h were exposed to LPS in the presence or absence of BAI for 6 h; immunofluorescent images of the HMGB1 (red) cytoplasmic translocation were observed with confocal scanning microscopy. Scale bar: 10 *μ*M. (g, h) HMGB1 levels in the cytoplasmic and nuclear fractions were analyzed by western blotting. GAPDH and LaminB1 were used as loading controls for cytoplasmic and nuclear fractions, respectively. All the results are presented as means ± SEM of three independent experiments. ^∗^*P* < 0.05, ^∗∗^*P* < 0.01, ^∗∗∗^*P* < 0.001, control vs. LPS or LPS+siRNA-1/2; ^#^*P* < 0.05, ^##^*P* < 0.01, BAI+LPS vs. LPS; ^&^*P* < 0.05, BAI+LPS vs. BAI+LPS+siRNA-1/2.

To further clarify that the BAI-modulated downregulation of HMGB1 release was attributed to the increased expression of SIRT1, we manipulated the expression and activity of SIRT1 using siRNA. SIRT1 was knocked down in BV2 cells by siRNA-1 or siRNA-2 transfection (Figure 7(a)). Similar to the *in vivo* results, the protective effect of BAI on microglia against LPS exposure was abolished in siRNA-1 or siRNA-2-transfected BV2 cells, indicating that BAI could no longer reverse the upregulation of HMGB1 release in LPS-treated cells (Figures 7(b) and 7(c)). We further analyzed the subcellular localization of HMGB1 and the expression of acetylated HMGB1 (Figures 7(d)–7(h)). The immunoblot analysis indicated that SIRT1 knockdown in cultured siRNA-1 or siRNA-2-transfected BV2 cells incubated with BAI and LPS resulted in higher acetylation of HMGB1 (Figures 7(d) and 7(e)) and enhanced nucleus-to-cytoplasm translocation (Figures 7(g) and 7(h)). Moreover, the subcellular localization of HMGB1 was confirmed by immunofluorescence staining (Figure 7(f)), which showed that BAI-inhibited HMGB1 release under LPS stimulation was abrogated by SIRT1 silencing.

## 4. Discussion

**Figure 8:**
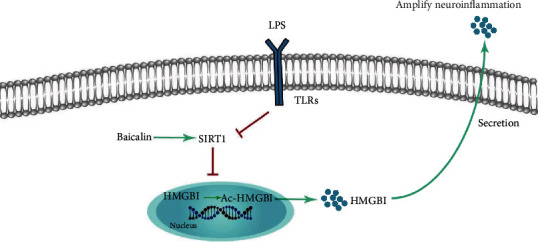
Schematic illustration of the possible protective mechanism of BAI administration in LPS-induced HMGB1 release in microglia cells.

The current study was conducted to examine the effects of BAI on cognitive dysfunction induced by a single systemic dose of LPS injection in mice and assess the changes in neuroinflammation and behavior. We found that BAI improved cognitive dysfunction induced by intraperitoneal administration of LPS. The protective effects of BAI on cognitive impairment in mice appeared to be related to its anti-inflammatory activity in microglial cells, modulating the activity of SIRT1, as well as downregulating the HMGB1 signaling pathway (Figure 8).

Several studies have shown that LPS-induced systemic inflammation impaired learning and memory [7, 8, 32] through increasing glial cell activation, production of proinflammatory mediators, and A*β* accumulation. Notably, LPS-induced inflammation and pattern recognition receptors expressed at the BBB surface can lead to endothelial inflammation and subsequent neuroinflammation [6]. Single systemic LPS-induced cytokines and alarmins in the serum also directly disrupt the BBB and alter BBB function, including adsorptive transcytosis, immune cell trafficking, and various transport functions, thus allowing proinflammatory immune cells and molecules to the CNS [42, 43]. Neuroinflammation is characterized by the activation of CNS resident microglia and expression of proinflammatory mediators, and is a pivotal feature in virtually every neurological cognitive impairment [6]. Indeed, reactive microglia can induce cytokine release and astrocyte activation [44], which leads to inflammatory toxicity and neuronal cell death [6]. Furthermore, the activity of astrocytes, another important type of glial cells in the brain, may exacerbate inflammatory reactions and tissue damage. Age, trauma, infection, and autoimmune response increase the number of astrocytes and induce rapid synthesis of GFAP [45]. Our results demonstrated that BAI mitigates LPS-induced increase of Iba-1 and GFAP and participates in the modulation of microglia and astrocyte activations, which is consistent with previous studies [21, 29]. Activated microglia are the main cellular source of central proinflammatory cytokines; hence, we focused on microglia in this study. By the same token, we used systemic LPS injection into rodents as it has been extensively used as a model for studying the interaction between inflammation and cognitive deficits.

BAI is an FDA-approved medical food flavonoid. Numerous studies have demonstrated that some natural phytochemicals have neuroprotective effects including on learning and memory impairment [46]. BAI has been assessed for safety, toxicity, and tolerability properties in *in vivo* and *in vitro* studies [47]. Thus far, a wide array of neuroprotective effects of BAI on neurodegenerative diseases such as ischemic stroke, Alzheimer's disease, Parkinson's disease, and traumatic brain injury have been reported by different studies. In addition, BAI exhibits considerable anxiolytic-like and antidepressant effects. It has been revealed that BAI has several pharmacological activities, including antioxidant, anti-inflammatory, antiapoptotic, and antiexcitotoxicity properties [21]. BAI and its metabolites have been shown to induce SIRT1 expression and activation [12]. Herein, we examined the potential of BAI as an agent for LPS impairment therapy. In terms of results obtained in this study, both LPS-induced injury and the protective effect of BAI may play a dual role in the periphery and CNS. To elucidate the roles of SIRT1 in the BAI protective effects on LPS-impaired microglial action and cognition, we used SIRT1 inhibitor EX-527 in LPS-induced mice. In the absence of SIRT1, the BAI effects on cognition dysfunction and neuroinflammation were largely attenuated. These results were further confirmed in the *in vitro* experiments with SIRT1 siRNA in microglial BV2 cells.

SIRT1 is an NAD-dependent deacetylase mainly involved in stress responses, cellular metabolism, and aging [9]. Its levels in the brain, especially in the hippocampus, are obviously higher than those in other tissues in mammals [11]. Compelling evidence has indicated that SIRT1 plays an important role in neurodegenerative diseases and cognitive dysfunction [48–50]. Furthermore, SIRT1 inhibits NF-*κ*B signaling activation and the resultant production of proinflammatory cytokines and protects neuronal PC12 cells from caspase-3-dependent apoptosis [49]. SIRT1 activation by resveratrol has been found to inhibit matrix metalloproteinases, regulate BDNF signaling, and reduce the production of a wide range of cytokines [48, 51]. Consistently, we demonstrated here that the level of SIRT1 protein was dramatically decreased in microglial cells in the hippocampus after LPS exposure, and this effect was reversed by BAI. In addition, SIRT1 is one of the downstream pathways of adenosine monophosphate-activated protein kinase (AMPK) activation. AMPK increases the intracellular NAD^+^/NADH ratio to activate the NAD^+^-dependent type III deacetylase SIRT1 [52]. BAI attenuates hyperglycemia aggravated ischemia/reperfusion injury in a manner dependent on AMPK [53]. Therefore, we think that BAI may also upregulate SIRT1 in an AMPK-dependent manner in the current study.

The interaction between SIRT1 and HMGB1 has previously been documented during an inflammation response. HMGB1 is a novel deacetylation target of SIRT1 [54], followed by acetylation, nucleus-to-cytoplasm translocation, and release from stressed cells, unleashing a signaling cascade of events leading to neuroinflammation. It is widely accepted that HMGB1 is a key inflammatory mediator in the pathogenesis of cognition dysfunction. Microglial activation and the subsequent inflammatory response in the hippocampus can disrupt neuronal networks required for learning and memory. Reportedly, acetylation of HMGB1 is a key process prior to its nucleus-to-cytoplasm translocation and extracellular secretion from kidney cells, accelerating the development of sepsis-associated acute kidney injury (SA-AKI) [55]. In the present study, we found that LPS-mediated SIRT1 inhibition increased cytosolic HMGB1 levels, while BAI-mediated SIRT1 activation facilitated HMGB1 translocation from the cytoplasm to the nucleus in BV2 cells. Thus, the exact roles of the SIRT1/HMGB1 pathway and its regulatory roles in microglial dysfunction and dNCR are of great importance.

There are several limitations in the current study. First, we did not measure both intracranial and peripheral cytokine levels at the same time in this study. Second, we only explored the role of SIRT1/HMGB1 signaling in microglial cells. In future studies, the role of this signaling pathway in other cell types such as neurons and astrocytes should be investigated. Finally, our study focused on relatively short-term cognitive functions (4–5 days after LPS exposure), and the long-term effects of BAI pretreatment remained unexplored.

In summary, we uncovered the SIRT1/HMGB1 pathway by which BAI prevents LPS-induced cognitive dysfunction and neuroinflammation. Defining the detailed mechanisms by which activation of microglial SIRT1 and degradation of HMGB1 protein-mediated the cognitive-improving effect of BAI may indicate a promising therapeutic target for the treatment of dNCR.

## 5. Conclusions

Our study demonstrated that BAI can improve acute neurocognitive impairment and inhibited neuroinflammation in LPS-induced mice *via* a SIRT1-dependent pathway and involved the inhibition of HMGB1. These data confirmed that BAI may have efficient medicinal value and proposed it as a new potential candidate for dNCR therapy (Figure 8).

## Data Availability

The data used to support the findings of this study are included within the article.
